# Alpha-Synuclein in the Gastrointestinal Tract as a Potential Biomarker for Early Detection of Parkinson’s Disease

**DOI:** 10.3390/ijms21228666

**Published:** 2020-11-17

**Authors:** Dominika Fricova, Jana Harsanyiova, Alzbeta Kralova Trancikova

**Affiliations:** 1 Institute of Neuroimmunology, Slovak Academy of Sciences, 814 38 Bratislava, Slovakia; dominika.fricova@savba.sk; 2 Department of Pathophysiology, Jessenius Faculty of Medicine in Martin, Comenius University, 814 99 Bratislava, Slovakia; jana.harsanyiova@uniba.sk; 3 Biomedical Center Martin, Jessenius Faculty of Medicine in Martin, Comenius University, 814 99 Bratislava, Slovakia

**Keywords:** Parkinson’s disease, alpha-synuclein, gastrointestinal tract, biomarker, oligomeric alpha-synuclein, PMCA, LCO, microRNA

## Abstract

The primary pathogenesis associated with Parkinson’s disease (PD) occurs in peripheral tissues several years before the onset of typical motor symptoms. Early and reliable diagnosis of PD could provide new treatment options for PD patients and improve their quality of life. At present, however, diagnosis relies mainly on clinical symptoms, and definitive diagnosis is still based on postmortem pathological confirmation of dopaminergic neuronal degeneration. In addition, the similarity of the clinical, cognitive, and neuropathological features of PD with other neurodegenerative diseases calls for new biomarkers, suitable for differential diagnosis. Alpha-synuclein (α-Syn) is a potential PD biomarker, due to its close connection with the pathogenesis of the disease. Here we summarize the currently available information on the possible use of α-Syn as a biomarker of early stages of PD in gastrointestinal (GI) tissues, highlight its potential to distinguish PD and other neurodegenerative diseases, and suggest alternative methods (primarily developed for other tissue analysis) that could improve α-Syn detection procedures or diagnostic methods in general.

## 1. Introduction

Parkinson’s disease is (PD) the second most common age-related neurodegenerative disease after Alzheimer’s disease (AD). PD affects approximately 1% of the population over the age of 60, with an increasing incidence with age [1,2]. Clinically, the disease is mainly characterized by the presence of motor symptoms, such as bradykinesia, postural instability, and resting tremor [3]. However, the presence of non-motor symptoms such as olfactory disorders, gastrointestinal (GI) dysfunction, autonomic failure, cognitive disorders, mental health problems, sleep disorders, pain, and excessive fatigue, not only accompany this disease, but many of them even precede the occurrence of motor symptoms [4,5]. It is the occurrence of non-motor symptoms, such as GI dysfunction, which occur up to 20 years before the onset of motor symptoms themselves [6,7], that provides a wide window in terms of early diagnosis, and thus potentially therapy for these patients. This is exacerbated by the fact that current therapy is based mainly on symptomatic or levodopa (L-DOPA) treatment but does not target the pathogenesis of the disease itself. Despite intensive efforts, the development of new and more successful therapeutic approaches has been insufficient, and fully dependent on early diagnosis of the disease. However, the diagnostics, especially in the early stages of the disease, currently mainly rely on very non-specific clinical symptoms, such as olfactory disorders, GI dysfunction, mood, and sleep disturbances. Whereas, the definitive confirmation of the disease is still based on a post-mortem pathological evaluation of the degeneration of dopaminergic neurons in the *substantia nigra pars compacta (SNpc),* and the presence of aggregated protein in a neuronal soma called the Lewy body (LB) and in neuronal processes called Lewy neurites (LN) [8,9,10]. Another complication in the diagnosis is the similarity of the clinical, cognitive, and neuropathological signatures of PD with other neurodegenerative diseases, such as PD with dementia (PDD), dementia with LB (DLB), AD, multiple system atrophy (MSA), or progressive supra nuclear palsy (PSP) [8,11,12,13,14,15,16]. Due to these similarities, up to 20–35% of patients are misdiagnosed with PD, while not meeting strict clinical or histopathological criteria [8,17,18,19]. For example, cognitive disorders that occur in 10–80% of cases of PD, especially in the later stages of the disease, significantly overlap with PDB, DLB, AD, and MSA [8]. However, in contrast to PD, in the case of DLB and PDD, motor symptoms appear concurrently or later, after the onset of cognitive impairment [20]. In the case of histopathological signatures, typical spherical LB and LN occur in PD, DLB, and PDD [20,21], while accumulated α-Syn in MSA is localized in oligodendroglia or glial cytoplasmic inclusions, without LB with typical spherical structures [22]. It is MSA that is often misdiagnosed as PD, especially in the early stages of the disease. However, the disease is more progressive compared to PD, and the response to dopaminergic treatment is weaker [23].

For these reasons, increasing efforts are focused on identifying biomarker(s) that would be able to detect PD in the early stages, as well as distinguish PD from other synucleinopathies. The ideal marker should meet several parameters, namely it should be (i) present in the early stages of the disease, (ii) consistent with the pathomechanism of the disease, (iii) have specificity and be able to differentiate similar diseases, (iv) reproducible, (v) easily available in tissues or body fluids, and (vi) cost effective for use in clinical practice [14,24].

### 1.1. Diagnostic Potential of α-Syn

α-Syn, as the major component of LB and LN, is consistent with the pathogenesis of PD and represents one of the possible candidates for a suitable biomarker [25]. In addition, mutations in *SNCA* gene (A53T, A30P, E46K, H50Q, G51D), its duplications, and triplications, have been found in familiar PD cases. These mutations are associated with the induction of oligomerization and fibrilization of α-Syn, and thus with the development of PD [20,26,27]. Similarly, polymorphism in *SNCA*, or its promoter, poses an increased risk of disease [28,29]. On the other hand, it should be borne in mind that LB are present in brainstem nuclei in about 8–12% of clinically asymptomatic populations over 60 years of age, which is thought to be a pre-symptomatic stage of the disease [8,30].

α-Syn is a small (140 aa), cytoplasmic protein, highly expressed in the brain, where it represents 1% of the total protein. This high expression in brain structures, as well as its localization predominantly in the neuronal presynaptic terminals, and the ability to interact with membrane structures, predetermines its function in modulating neuronal membrane stability, and regulating synaptic function, and vesicular transport [4]. In addition to brain structures, α-Syn is also present in body fluids (cerebrospinal fluid (CSF), plasma, blood) [8,26,31,32,33] and in peripheral tissues (skin, GI tract (GIT), olfactory glands, gonadal tissues) [14,34,35,36,37,38].

### 1.2. Structure, Posttranslational Modifications, and Toxic Forms of α-Syn

α-Syn is a cytoplasmic protein, naturally occurring as an unstructured monomer or metastable tetramer, which is able to adopt a wide range of dynamic structures, depending on the environment and binding partners [39,40,41]. Several different isoforms of α-Syn have been identified, including truncated variants (112, 126 and 98 aa) possessing a higher risk of pathological α-Syn aggregation [42].

**Figure 1:**
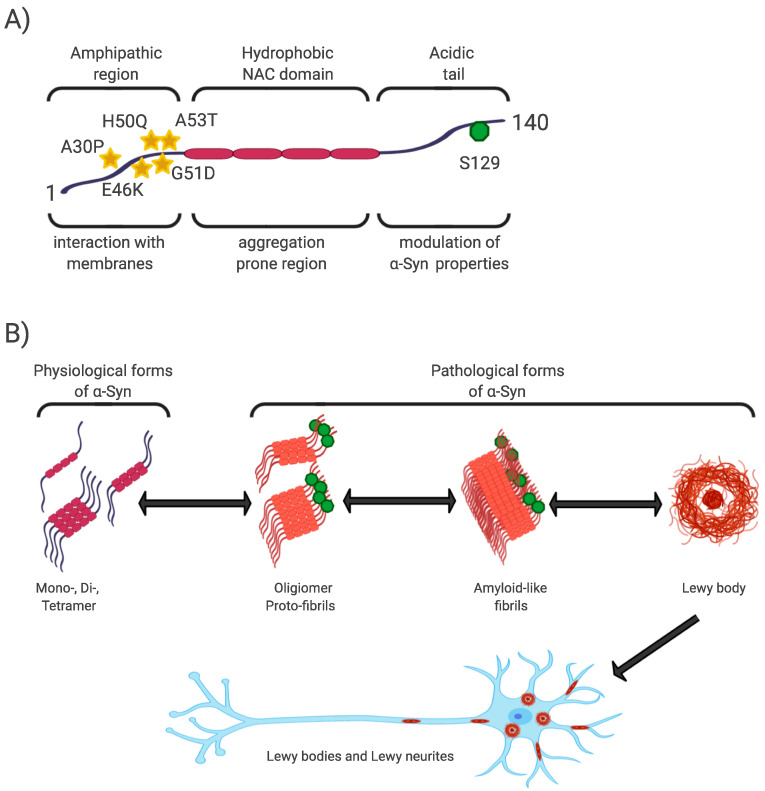
α-Syn structure, properties, and pathological forms. (**A**) Simplified scheme of α-Syn structure. α-Syn is a 140 aa protein, where the N-terminal and non-amyloid-β component (NAC) regions are responsible for its interaction with membrane structures. Mutations associated with Parkinson’s disease (PD) are located in the N-terminal region. The NAC region, the most hydrophobic part of the protein, is involved in random-coil conformational changes on the β -sheet. The unstructured C-region is responsible for the structural and physiological properties of the protein. The most discussed post-translational conditioning, phosphorylation at S129, is located in the C-terminal domain. (**B**) α-Syn is a cytoplasmic protein that occurs naturally as an unstructured monomer or metastable tetramer [49]. Under pathological conditions, its conformational changes occur from a random-coil to a beta-sheet structure to form unstable oligomers and protofibrils, followed by fibrils until insoluble protein aggregates are formed. These are localized in the neuronal soma (Lewy body) or in the neuronal processes (Lewy neurites).

The N-terminal and central regions of the protein (1–95 aa) are responsible for its interaction with membrane structures, whereas interaction with lipids is thought to play a role not only in modulating its function but also in changing its conformation from unstructured to highly organized fibrils [20,40,43]. In this regard, the central NAC region (non-amyloid-β component, 60–95 aa), as the most hydrophobic part of the protein, is able to undergo a conformational change from random coil to β-sheet structure, and form cylindrical β-sheets and amyloid-β-like fibrils [44,45,46]. It is the process of nuclear-dependent gradual α-Syn oligomerization and fibrilization that is considered critical in the development of PD, with the highest rates of cytotoxicity observed especially in unstable oligomeric or protofibrillar forms of α-Syn [20,26,47]. In addition, PD-associated point mutations are located only in the N-terminal region (A53T, A30P, E46K, H50Q, G51D) [48] (Figure 1).

The C-terminal region (96–140aa) is present in all conformational forms of the protein (monomeric, fibrillar) or in its membrane-bound form, and is responsible for the internally disordered nature of α-Syn [50,51,52,53]. It is involved in the modulation of α-Syn properties, such as interactions with other proteins [54,55,56], metal ions [57], and other ligands [45,58]. In addition, this area is rich in regulatory posttranslational modifications (PTM), which are likely to play an important role in the regulation of α-Syn structural and physiological properties [52,59] (Figure 1).

### 1.3. The Role of α-Syn Phosphorylation in the Development and Diagnosis of PD

Phosphorylation at S129 (p-S129) is the most discussed PTM in α-Syn, its role in modulating α-Syn physiological function and promoting or suppressing α-Syn toxicity is still unclear [52]. In vitro and in vivo studies have confirmed the importance of p-S129 in the regulation of: (i) the interaction of α-Syn with membrane structures, with p-S129 showing an inhibitory effect in this respect; (ii) protein–protein interactions; (iii) synaptic plasticity, although the mechanism remains unexplained; (iv) α-Syn nuclear translocation; (v) interactions with metal ions, thereby affecting metal ions-mediated α-Syn aggregation properties; and last but not least, (vi) turnover, where p-α-Syn is considered to represent a late stage in the pathogenesis of α-Syn, and its main role is to selectively target proteins for their degradation [52,59,60].

### 1.4. The α-Syn Strains

**Figure 2:**
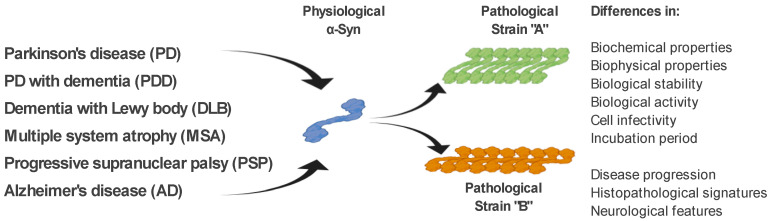
α-Syn strains and their discrimination. Under pathological conditions, different types of α-Syn aggregates (strains), with specific biochemical, biophysical, and biological properties, occur in the brain of patients with various neurodegenerative diseases. It is the different properties of these strains that are responsible for the different clinical manifestations of these diseases. Differential diagnosis of PD and related neurodegenerative disorders is currently quite challenging. We propose several methods to discriminate such disorders, based on the specific physical and biochemical properties of α-Syn strains.

As mentioned above, ß-sheet-rich α-Syn fibrils occur in several neurodegenerative diseases (PD, PDD, DLB, MSA, and AD). However, these diseases possess a different clinical manifestation as well as a pathological signature [61]. The fact that α-Syn is able to form several types of fibrillary structures (strains), with different conformational properties in vitro and different biological activities, suggests that various types of strains are present in the brain of patients with different neurodegenerative diseases, and probably responsible for various clinical manifestations of these diseases [61,62,63,64,65]. This is also supported by a recent study, where in transgenic TgM83 mice, various α-Syn strains, either *in vitro* generated or isolated from brain-derived aggregates, targeted different brain regions and cell types in these mice, but also induced different motor, pathological, and biochemical phenotypes [64]. In a more detailed characterization, using cell models but also transgenic animals, the authors were able to define different α-Syn strains based on a wide range of parameters, such as: biochemical properties, including resistance to protease degradation denaturation by chaotropic agents; biological stability; biological activity, including inhibition of proteasome activity; cell infectivity and incubation period; pathological lesion tropism; and neurological features in transgenic animals [64,66] (Figure 2).

## 2. The Role of miRNA in the Regulation of Expression and Aggregation of α-Syn

MicroRNAs (miRNAs) are endogenous single-stranded noncoding RNAs consisting of 17–24 base-pairs that function as important post-transcriptional regulators of gene expression. Furthermore, miRNAs have emerged as suitable tools for the diagnosis, prognosis, and even as targets for new therapeutical strategies for a variety of pathologies [67,68]. In this regard, miRNAs are also involved in the regulation of α-Syn, with several miRNAs identified that target α-Syn through direct interaction, or that contribute to changes in α-Syn levels and toxicity indirectly, involving various pathways regulating α-Syn clearance. As mentioned above, higher expression levels of α-Syn arising from *SNCA* genomic duplication or triplication are associated with familial PD [27,69,70]. Moreover, the regulation of α-Syn expression, degradation pathways plays a crucial role in the control of α-Syn intracellular levels, with mounting evidence supporting the role of various miRNAs in these key processes opening a new avenue for their investigation in relation to PD. In this context, there are already some clinical data suggesting that patients with PD have significantly lower levels of miR-7 in *SNpc* [71].

### 2.1. miRNAs Interacting Directly with α-Syn mRNA

All identified miRNAs that directly bind the 3′-untranslated region (UTR) of α-Syn mRNA negatively regulate its expression, and include miR-7, miR-153, miR-34b, miR-34c, and miR-214. The role of miR-7 as a negative regulator of α-Syn expression has been confirmed by several independent studies [72,73,74,75,76,77,78], using both in vitro and in vivo models for PD. In these studies, the authors demonstrated that overexpression of miR-7 increased cell survival and viability after 1-methyl-4-phenyl-1,2,3,6-tetrahydropyridine (MPTP) treatment [74,75], and that in an MPTP-induced mouse model of PD, the miR-7 was reduced and correlated with α-Syn upregulation in the *SNpc* [71,72,74]. In addition, several other direct targets of miR-7 have been identified, and multiple mechanisms of its neuroprotective role have been proposed including: (i) inhibition of inflammation [74,77,78,79]; (ii) direct activation of the mTOR protective pathway [75]; (iii) inhibition of apoptosis [80]; (iv) promotion of mitochondrial functions [81]; and (v) reduction of cellular oxidative stress [82]. It should be noted that the role of miR-7 has also been identified in the peripheral organs in mice, where overexpression of miR-7 in pancreatic β-cells resulted in a reduction of α-Syn mRNA [76]. Similarly to miR-7, miR-153 also downregulates α-Syn levels through direct interaction with its 3′-UTR [73]. It is likely that miR-7 and miR-153 are involved in different parts of the regulation of α-Syn levels, with an additive effect. MiR-7 is thought to modulate translational inhibition of α-Syn, when miR-153 was involved in the degradation of α-Syn mRNA [73]. Accordingly, overexpression of miR-153 reduced the MPTP-induced neurotoxicity in DA neurons [75]. Direct targeting of 3′-UTR of α-Syn mRNA by miR-34b and miR-34c has also been proved to inhibit its expression and resulted in a decrease of α-Syn aggregate formation [83]. miR-34c-5p was found to be decreased in the amygdala, frontal cortex, *SNpc*, and cerebellum of PD patients compared with a healthy control group [84].

### 2.2. miRNAs Indirectly Regulating α-Syn Levels

Several different pathways have been shown to have an indirect impact on the levels of α-Syn, and thus its aggregation and toxicity. The majority of them are involved in α-Syn clearance through either autophagy [85,86,87,88,89] or chaperone-mediated autophagy [90,91,92,93]. In the context of autophagy, mir-128 binds to transcription factor EB (TFEB), one of the key regulators of this process [92], with its miR-128-mediated suppression resulting in the accumulation of the α-Syn, and an increase in its toxicity in dopaminergic neurons [90]. Let-7 is another miRNA that presumably regulates α-Syn levels, through the formation of autophagosomes that lead to the failure of α-Syn lysosomal degradation [91,93]. In the case of chaperone-mediated autophagy, α-Syn is recognized by the chaperone heat shock protein 70 (Hsp70), which directs α-Syn into lysosome through its interaction with the membrane receptor, lysosomal-associated membrane protein 2a (Lamp2a) [87]. When Hsp70 or Lamp2a are downregulated, α-Syn accumulates [85,88], wherein multiple miRNAs target the 3′-UTR of Hsp70 (hsa-miR-26b, hsa-miR-106a and hsa-miR-301b) or 3′-UTR of Lamp2a (hsa-miR-379). Importantly, the analysis of brains from PD patients confirmed almost all identified miRNAs were significantly increased in the *SNpc* [86].

## 3. Diagnostic Potential of Gastrointestinal Tract Tissues

Regarding the material itself suitable for diagnostic analysis, several studies have focused on body fluids (CSF, plasma, serum, saliva) or peripheral tissues (GIT, salivary glands, skin, olfactory mucosa, gonadal tissues) [8,14,94,95]. Despite considerable efforts to use body fluids as potential diagnostic material, due to their relatively easy availability, or the fact that they offer many advantages over clinical options, their sensitivity and specificity are insufficient to diagnose or predict PD [94,96,97]. Therefore, attention has increasingly focused on peripheral tissues. In terms of specificity and sensitivity, the detection of pathological aggregated forms of α-Syn in the GIT or salivary glands appears to be a better option compared to the skin or olfactory mucosa [94]. Findings that non-motor symptoms are observed several years before the onset of motor symptoms themselves, as well as the fact that almost 60% of patients suffer from constipation [37], also favor GIT as a suitable tissue for the early diagnosis of PD.

To date, studies have focused on confirming the presence of protein inclusions or pathological forms of α-Syn and neurodegeneration in the tissues of PD patients, as the only direct evidence of PD-related neuropathological changes in the peripheral nervous system [34]. Since the first mention of the presence of LB in the GIT in 1984 [98], an increasing number of studies have focused on post-mortem autopsy studies of peripheral tissues as a promising material and method for accurate detection of PD-associated neuropathological changes.

The enteric nervous system (ENS) is a complex neural network localized in the wall of the GIT [99], where it controls physiological functions, such as motility, secretion of mucosal fluids, and blood flow in the GIT by complete reflex circuits [100,101]. The ENS, located in the tubular alimentary tract, includes neuronal cells bodies, axons and enteric glial cells, forming interconnected networks whose cell bodies are concentrated in the ganglia of two main plexuses: Meissner’s (submucosal) plexus and Auerbach’s (myenteric) plexus. The myenteric plexus is present between muscle layers in the whole digestive tract, from proximal esophagus to the internal anal sphincter [99,100]. The submucosal plexus is situated especially in the small and large intestines, even though independent submucosal ganglia are found in the esophagus or stomach without creating interconnected plexus.

The first studies using autopsies to map Lewy pathology in GIT tissues in patients with histologically confirmed PD in various parts of the brain were conducted as early as the 1980s [98,102,103]. Qualman with colleagues (1984) observed LB in the esophageal myenteric plexus of one PD patient, and the colon of another patient with PD. Later Wakabayashi et al. (1988) focused on Lewy pathology localized in the myenteric and submucosal plexuses of the entire GIT of seven PD patients, but LB were also detected in eight out of 24 healthy individuals. Of note, in these studies, the authors detected the LB using haematoxylin and eosin staining, without α-Syn detection.

Pathologically aggregated, phosphorylated (p-α-Syn), filamentous αS (f-α-Syn), or Lewy pathology have been found in autopsies and biopsies of PD patients in both the upper and lower GIT. Several studies have shown that the distribution of α-Syn forms in the GIT of PD patients occurs with a rostrocaudal gradient [102,104,105,106], except for the proximal esophagus [104]. This fact can be explained by the very nature of the vagal innervation in the GIT, which is widely and numerically distributed from the esophagus to the stomach, and its concentration further decreases from the small intestine to the proximal colon. Thus, α-Syn pathology observed in GIT originates more from vagal innervation than ENS or sympathetic innervation. Moreover, the cell bodies innervating the upper part of the esophagus are located in the nucleus ambiguous, while the rest of the GIT is innervated by neuronal cell bodies derived from the dorsal motor nucleus of the vagus nerve [104].

### 3.1. The Presence of Total α-Syn in the GIT Tissues

Several studies have focused on the detection of total α-Syn (t-a-Syn) in various GIT tissues, where the authors observed t-α-Syn-positive inclusions across the entire GIT. Number of studies have shown the high specificity of t-α-Syn in autopsy or biopsy samples [34,105,107,108], and pointed to the diagnostic potential of t-α-Syn. For example, as early as 2006, Braak and colleagues observed t-α-Syn-positive inclusions throughout the gastric ENS, including both plexuses as well as peripheral nerves of the adventitia in the autopsy specimens of patients with sporadic PD (5/5), but not in healthy individuals (0/5) [105]. A similar profile was also observed in gastric mucosal biopsies of patients with PD (17/28 patients), pre-motor PD (1/6), while t-α-Syn-positive inclusions were present in only 1/23 of control samples. The authors claimed 85% sensitivity and 95.2% specificity in this study [107]. Due to minimal invasiveness, the availability of tissue samples, minimal side effects of the procedure, and the possibility of using local anesthesia, biopsies obtained from salivary glands have become a promising candidate, and a potentially suitable tissue biomarker to demonstrate α-Syn pathology in the premotor stage PD. However, the presence of t-a-Syn-positive inclusions was confirmed in autopsy and biopsy samples of patients with PD (3/3), while, as in previous studies, control samples did not show positivity (0/3) [34,35].

The colon has become the most studied part of the digestive tract in relation to PD [109]. Shannon and colleagues observed the presence of t-α-Syn in colonic biopsies of pre-motor PD patients (9/9), in contrast to control samples (0/3) [108].

Contrary to these data, several studies point to the presence of t-α-Syn in colonic biopsy specimens not only in PD patients, but also in control samples [110,111,112]. In this regard, Gold and colleagues used biopsies of the colonic mucosa of 10 PD patients and 77 healthy individuals. All PD samples were positive for t-α-Syn, but 52% of the samples from healthy individuals were also positive for t-α-Syn. On the other hand, the results revealed significantly higher t-α-Syn expression in PD patients compared to healthy individuals. Very similar results were reported in a recent study in which the authors analyzed gastric and colon biopsies from pre-motor PD patients (7) and controls (18). Immunoreactivity for t-α-Syn in the enteric and colon tissues of the myenteric plexus as well as the submucosal plexus was detected in all PD samples. However, very high positivity was found in either the myenteric (82%) or submucosal (100%) plexus in the control samples [113]. In an interesting study, the authors compared not only samples of colonic mucosal biopsy of patients with PD in the early (15) and late (7) stages of the disease with samples from healthy individuals (11), but also detection methods, i.e., classical in most cases, and used immunohistochemical analysis (IHC) and paraffin-embedded (PET) tissue blot. Both methods showed high specificity for the detection of t-α-Syn (80–100%), which, however, was at the same level in the control samples (80–100%). It should be noted that the authors focused on the proximal part of the GIT, i.e., the sigmoidal colon and rectum [112]. Based on these results, which are quite inconsistent, especially in the positivity of control samples, the authors do not recommend t-α-Syn as a suitable biomarker of early stages of PD.

### 3.2. Phosphorylated α-Syn in the GIT Tissues

Since t-α-Syn can reflect both physiological and pathological forms of α-Syn, several authors have focused more on its pathological forms, and specifically on the p-129 phosphorylated form. Although it does not directly define probably the most toxic oligomeric forms of α-Syn or f-α-Syn, due to the fact that p-α-Syn S-129 is considered a late stage in the pathogenesis of α-Syn, its detection might be considered a suitable diagnostic marker.

Several studies have reported the presence of t-α-Syn, as well as p-α-Syn, not only in PD patients, but also in PDD, DLB, and AD. In patients with PD, p-α-Syn was detected mainly in the upper GIT, such as the submandibular glands and distal esophagus, with the incidence of p-α-Syn gradually decreasing in the rostrocaudal axis from the stomach through the small and large intestine to the rectum [106,113]. As already mentioned, the salivary glands could be a promising type of tissue for the early detection of PD-related pathology. Regarding p-α-Syn, positivity was present in submandibular gland samples in 9/12 PD patients [114], as well as in bilateral submandibular needle biopsies in six premotor and one advanced patient with PD [115]. The density of p-α-Syn varied over time, with a specific increase reported in five subjects, one unchanged, and a decrease found in one subject. The limitation of these studies was that control individuals were not included, and the number of PD patients was not sufficient. As dysphagia is considered a serious PD symptom that is a major cause of death in PD patients, the pharynx has also become a focus for the detection of pathological forms of α-Syn [116,117]. Aggregated p-α-Syn was observed in the pharyngeal motor [117] and sensory [116] nerves, innervating the pharynx in PD patient samples in contrast to control samples. In addition, the density of p-α-Syn aggregates was higher in PD patients with dysphagia compared to patients without dysphagia, suggesting a direct association between pharyngeal sensory nerves and pathological PD processes.

In contrast to colon studies aimed at detecting the t-α-Syn, p-α-Syn analyses have more informative value, also due to the larger groups of patient and control used. Several studies have observed p-α-Syn positive LN from 72% (21/29) [37] to 74% (23/31) [118] in colonic biopsy specimens from PD patients, where none of the control specimens did not show p-α-Syn positivity. Consistent with these results, the largest study to date focused on the detection of pathological forms of α-Syn in the GIT, where the authors observed p-α-Syn in mucosal and submucosal neurites only in 7/60 pre-motor PD patients, but none was present in 0/161 healthy individuals. Of note, the authors analyzed not only colonic material, but the entire GIT tissues, with p-α-Syn positivity also present, in addition to the colon, in gastric and duodenal samples. Esophageal samples did not show any positivity [119]. However, even these studies are not entirely unambiguous, some authors also describe different results in the case of detection of p-α-Syn in colon biopsies, mainly in terms of high positivity in healthy individuals [112,120,121].

Interestingly, a recent analysis of p-α-Syn in gastric and colon samples of pre-motor PD patients (7) and control samples (18), revealed deposition of p-α-Syn aggregates in the propria muscularis in 100% of pre-motor PD patient samples, but in the Meissner’s plexus from only one patient. This study therefore suggests the detection of p-α-Syn (but not t-α-Syn) in the muscularis propria instead of submucosa or mucosa of the GIT tissues as a sensitive prodromal biomarker for PD. However, a larger group of patients with pre-motor PD and control subjects is necessary for a suitable outcome.

Taken together, these results confirm the promising potential of GIT tissues for early detection of the disease, but still do not provide a clear answer to the question of which method and which tissue is most suitable in this regard. Based on the rostrocaudal distribution of t-α-Syn and p-α-Syn, as well as the vagal innervation of the organs, it is believed that proximal tissues are more suitable compared to distal tissues, such as the sigmoid colon and rectum. Detection of p-α-Syn shows higher specificity and thus a lower degree of positivity in control samples, compared to t-α-Syn. However, the limitations of the studies should not be forgotten: (i) most studies were based exclusively on IHC detection of p-α-Syn; (ii) the sensitivity and specificity of the methods tested are still inconsistent and debatable; (iii) small groups of PD patients or controls; (iv) patients at different stages of the disease, making it difficult to compare the obtained results; and (v) inconsistency in the choice of the GIT tissue and corresponding tissue layer.

Therefore, there is a need for the constant search and optimization of more suitable, sensitive, and specific methods for the detection of α-Syn as a biomarker of early stages of PD, as well as the unification of protocols in material collection and subsequent analyzes.

## 4. Alternative Methods and α-Syn Strains Discrimination

More and more studies have focused on the development of new methods for the detection of α-Syn, or the adaptation of various methodological procedures developed in priority for the detection of specific markers in other tissue types. Several studies have tested different methodological approaches for a suitable detection of α-Syn pathology in the colon of PD patients and healthy individuals [110,121,122]. However, neither different immunohistochemical methods, using different antibodies, showed specificity and sensitivity higher than 80% [110], nor standard biochemical analysis by 1-D or 2-D electrophoresis, showed differences in α-Syn expression levels, phosphorylation and aggregation between PD and control samples [121].

Nanoparticle-based methodologies, including sensor-based techniques, could also represent an interesting approach to early diagnosis of PD [123,124,125]. These methodologies are able to target the α-Syn oligomers with very high sensitivity up to the level of 1 fM [123]. In most cases, these methodologies have been used on recombinant systems, but information on the validation of these methodologies on human samples, such as human serum, is gradually emerging. It will be interesting to follow the development in this area, the results comparing control subjects and patients with PD at different stages of the disease, as well as the possibilities of using these techniques, for example in the tissues of the gastrointestinal tract.

### 4.1. Assays Based on Seeding Properties of α-Syn

One method of detecting pathological forms of α-Syn (oligomers (o-α-Syn) and f-α-Syn) utilizes their ability to nucleate and induce α-Syn aggregation. Cyclic amplification of misfolding proteins (PMCA), conceptually analogous to DNA amplification by PCR, was originally developed to detect pathological forms of prion protein [126]. PMCA is based on nucleation using oligomeric or fibrillar forms of the protein, called “seeds”, in the presence of an excess of monomeric protein. After several cycles, when the newly formed fibrils are repeatedly broken forming new “seeds”, and with their subsequent polymerization, the number of seeds increases exponentially. For detection, it uses sensitive and specific measuring of the fluorescence of Thioflavin T (ThT), a standard amyloidogenic fluorescent probe [127].

Fenyi and colleagues used this assay to detect the pathological form of α-Syn in antrum colon and rectal sigmoid biopsies from PD patients and healthy individuals and compared this method with conventional immunohistochemical staining using an anti-β-α-Syn antibody. In addition to the promising results regarding the specificity of the method, they also confirmed its higher sensitivity compared to IHC. In addition, the authors confirm the unsuitability of rectal biopsies as a material for the detection of α-Syn due to the insufficient amount of aggregated α-Syn in this area [122]. PMCA was also successfully used in CSF samples from PD patients and controls, including patients with other neurological and neurodegenerative diseases, where the authors correctly identified patients with PD with an overall sensitivity of 88.5% and a specificity of 96.9%. In addition, their results correlated with the severity of clinical symptoms of PD [128]. In the following study, the authors highlighted the potential of PMCA to discriminate PD and MSA with an overall sensitivity of 95.4%, and further confirmed that aggregates amplified from CSF had properties similar to those from the brain. Similarly, another test based on the principle of PMCA, real-time quaking-induced conversion (RT-QuIC), using o-α-Syn as a seed [129], demonstrated in the analysis of a CSF sample from patients with PD, DLB, AD, and healthy individuals, 93% diagnostic sensitivity and 100% specificity [130].

### 4.2. Assays Based on IHC

The majority of previous studies have been based on IHC using antibodies against t-α-Syn or p-α-Syn. However, there is an increasing effort to develop, characterize, and validate conformationally specific antibodies that better reflect pathological forms of α-Syn. Recently a detailed study was published in which the authors characterized 16 α-Syn conformation-specific antibodies using several biochemical methods. Although the authors suggest that most of the tested antibodies show high specificity for o-α-Syn and f-α-Syn, they point out that neither is able to distinguish between the two forms [131]. Nevertheless, their high specificity for pathological forms of α-Syn gives them a high potential for use as a potential diagnostic tool.

Higher IHC specificity could also be achieved using a variety of fluorescent amyloid ligands, such as conventional Congo red and thioflavins, or even luminescent conjugated oligothiophenes (LCO), which have been successfully used to discriminate prion strains [132]. However, LCOs detect a larger spectrum of aggregates compared to conventional amyloid ligands [133,134]. The nature of the fluorescence emitted from a given LCO is defined by the conformation of its thiophene chain, which in turn depends on the conformations of the assemblies to which it binds. Strain discriminative abilities of LCO were indicated in a study where the authors distinguished distinct α-syn conformers in the brain from patients with PD and MSA in the IHC frozen section. The strength of the LCO was further increased by a combination of advanced microscopy fluorescent lifetime imaging (FLIM), where the lifetime of the emitted fluorescence is characterized by a fluorophore environment which due to different fluorescence lifetimes allows detection of even very small changes (conformational changes of α-Syn) [135,136].

The proximity ligation assay (PLA) is based on IHC but is able to detect anti-protein–protein interactions, or protein complexes, in the case of PD, mainly o-α-Syn. In one study, the authors tried to use the potential of PLA to detect o-α-Syn in biopsy samples from different parts of the GIT from prodromal and advancer PD patients and healthy individuals but failed to distinguish prodromal from advanced PD samples or healthy controls. In addition, the authors tried to compare PLA with IHC also using, in addition to anti-α-Syn conformationally specific antibody, a PET blot that focuses on f-α-Syn. However, even with these methods, they failed to observe differences between groups [137].

### 4.3. Enzyme-Linked Immunosorbent Assay (ELISA)—Based Assays

ELISA is widely used in the detection of physiological as well as pathological (p-α-Syn, o-α-Syn) forms of α-Syn, especially in CSF, blood, plasma, and saliva samples in patients with PD [14,20,26,138], but adaptation of these methods to tissue sample analysis is not excluded. New methods such as Luminex, a derivative of an ELISA based on beads for binding antibody, or electro-chemiluminescence (ECLIA)-based multiplex assay, based on amplification of the detected signal by electro-chemiluminescent labels (SULFO-TAGs) bound to antibodies, have been developed to increase the sensitivity and specificity in detection of pathological forms of α-Syn in body fluid samples. Both assays achieved higher sensitivity (up to 9 pg/mL) [139,140] compared to ELISA (10–20 pg/mL) [138], but the obtained results from PD patients compared to healthy individuals are highly controversial, with none of the methods showing a specificity higher than 69–85% [8,140,141,142]. For example, in the case of the Luminex assay, a significant positive correlation between p-α-Syn concentrations and PD severity was confirmed, but the test showed diagnostic potential only when combined with t-α-Syn concentrations in CSF [48,140]. These results are also in line with the considerations of several authors, who instead suggest detecting not only t-α-Syn, but focusing on the ratio between o-α-Syn/t-α-Syn, or p-α-Syn/t-α-Syn, which could contribute to higher specificity of the method [14,138,141,143].

### 4.4. Other Biochemical Methods and α-Syn Strain Discrimination

Another group of methods utilizes the biochemical properties of aggregated α-Syn, namely resistance to cleavage by Proteinase K (PK), which cleaves physiological, monomeric α-Syn, but also reveals epitopes in the case of aggregated forms of α-Syn. In this way, it is possible to increase not only the specificity, but also the sensitivity, in the detection of pathological forms of α-Syn [144]. Methods such as classic western blot or paraffin embedded tissue (PET) blot in combination with PK digestion have been successfully used to discriminate prion strains [145].

As mentioned above, Shahnawaz with colleagues confirmed the potential of the PMCA assay to discriminate PD and MSA on CSF samples. However, they also focused on western blot analysis of PMCA-derived α-Syn fibrils from these samples, after PK cleavage, where they confirmed a specific migration profile for CSF-derived α-Syn fibrils from patients with PD and MSA. Migration profiles were also compared with the profiles of patient-derived fibrils, thus confirming the equivalence of α-Syn aggregates found in the brain or CSF of patients with distinct neurodegenerative diseases [65]. These results suggest that the same, disease specific α-Syn migration profile is likely to be present in GIT tissues as well.

PET-blot uses in situ protein detection and anatomical histoblot resolution on paraffin-embedded tissue samples. Cleavage of PK, in combination with PET-blot to detect the aggregated forms of α-Syn, was also used in the analysis of colonic mucosal biopsies from patients with early PD, advanced PD, and healthy controls, but despite the great potential of this method and 86% sensitivity, the results indicated very low specificity due to the positive signal detected in the control samples [137]. On the other hand, using the DLB brain samples, PET-blot revealed synaptic α-Syn-positive microaggregates in cortical and subcortical grey matter, with a higher sensitivity compared to classical IHC [146], which gives hope that the optimization of PET-blot technique could also lead to sensitive and specific detection of α-Syn aggregates in GIT tissues.

### 4.5. miRNA as a Biomarker for PD

miRNAs profiling is a novel and rapidly evolving direction in efforts to identify new biomarkers. Despite the currently variable results, an increasing number of studies have suggested a broad spectrum of miRNAs as potential biomarkers of PD in plasma or CSF [147,148,149]. Similarly, to the studies described above, in addition to certain technical aspects, variability in outcomes results from clinical differences between patients, such as disease stage, age, and comorbidity, but also from the sample itself (plasma, CSF) as sources of analysis [150].

Another variability in the analysis and identification of miRNAs as a suitable biomarker of disease, in general, results from the differential expression of miRNAs in tissues and circulating miRNAs in body fluids [151]. These differences result from the fact that changes in the expression of specific miRNAs are more pronounced at a particular site of pathology, for example in *SNpc* in PD patients [152]. Thus, in the case of PD, the presence of miRNA in serum or plasma is very limited due to the blood–brain barrier [153], and therefore may not reflect miRNA expression in tissues [151]. For these reasons, analysis of miRNAs directly from affected tissues appears to be a more suitable alternative. In line with these claims and given that miRNAs are also present in GIT and ENS tissue, a recent study clearly shows the potential of miRNA screening for identifying early stages of the cohort. Using the A30P mouse model of PD, the authors monitored not only functional (behavioral and physiological) histopathological but also molecular changes in the ENS (several parameters including behavioral and physiological functions (gastrointestinal contractions)), and histopathological changes in order to identify biomarkers in the gut related to early stages of PD [68]. In this mouse model, they were able to identify a whole panel of intestinal biomarkers of early PD, including miRNAs regulating α-Syn properties. Moreover, 31 miRNAs dysregulated in this PD model were also confirmed to be dysregulated in body fluids and brain tissues of patients with developed PD. Therefore, the results should also be confirmed in GIT tissues in patients in the early pre-motor stages of the disease. Collectively these data suggest a good potential for minimally invasive GIT biopsy and miRNA screening as one of the diagnostic methods for early detection of PD [68].

## 5. Conclusions

Despite enormous efforts to develop a suitable diagnostic method for the early stages of PD, or to find a suitable diagnostic marker, we still do not know the definitive answer to the questions: (i) which tissue or body fluid would be suitable for early detection of the disease; (ii) what methods are the most sensitive and appropriate; and (iii) whether α-Syn is an ideal biomarker for PD. There has been, and continues to be, a great deal of interest in the study of body fluids as a suitable material, mainly due to the relatively easy availability and proven methodologies for this type of analysis, but the results obtained so far are contradictory.

Therefore, and also due to the fact that GIT disorders are one of the first symptoms of PD, much of the interest has shifted to the study of GIT tissues. α-Syn has very promising preconditions for an important biomarker, due to its connection with the pathogenesis of the disease, the presence in the early stages of the disease, as well as the presence not only in the brain but also in peripheral tissues.

Here we summarized the existing knowledge about the presence of pathological forms of α-Syn in various organs of the GIT. However, most studies relied solely on IHC detection, many groups of patients were not large enough, studied tissues differed not only in the organs themselves, but also in the individual layers of these organs (e.g., myenteric plexus, mucosa) leading to controversy in the obtained results. Nevertheless, we can say that due to rostrocaudal distribution α-Syn, the distal parts of the GIT (sigmoid colon and rectum) are probably not suitable as a material for early detection of the disease. As for the forms of α-Syn themselves, from the studies we are inclined to believe that the detection of t-α-Syn is not entirely appropriate because it reflects the presence of, not only pathological, but also physiological, forms of α-Syn. It is the focus on p-α-Syn, o-α-Syn, or f-α-Syn, or on the ratio between o-α-Syn/t-α-Syn, or p-α-Syn/t-α-Syn, that could lead to a higher specificity of methods.

**Figure 3:**
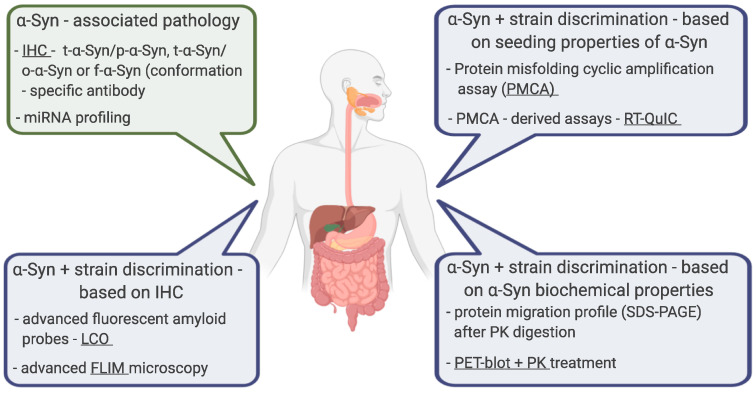
Alternative methods using gastrointestinal tract (GIT) tissues to detect α-Syn related pathogenesis and its strains discrimination. We propose a conventional immunohistochemical analysis (IHC) focused on the detection of pathological forms or the ratio between t-α-Syn and these forms instead of the detection of t-α-Syn. Similarly, miRNA profiling targeting specific disease-modulating miRNAs might be a relatively rapid tool for the detection of the early stages of the disease in GIT tissues (green boxes). Because different α-Syn strains are responsible for the diversity of clinical manifestations of neurodegenerative diseases, we propose several methods to distinguish these strains based on their specific physical and biochemical properties (blue boxes).

In addition, we tried to highlight the potential of α-Syn to discriminate against various neurodegenerative diseases, either using advanced fluorescence probes (LCO) and microscopy (FLIM), using strain-specific seeding properties of α-Syn (PMCA), or in combination with strain-specific biochemical properties of α-Syn (PK digestion). We also wanted to remind about the hitherto little-explored potential of miRNAs in the diagnosis of early stages of PD, emphasizing the presence of these miRNAs in the gut, and thus representing potential complementary and indirect targets for the analysis of α-Syn in the GIT in the future (Figure 3).

As mentioned above, these methodologies currently do not achieve the required sensitivity and specificity, due also to the lack of recognition of suitable diagnostic material, inconsistencies in the analyzed samples, as well as differences in the protocols of individual procedures. However, in recent years we have witnessed not only progress, but also a relentless effort, to improve these methodologies, for example by using conformational specific antibodies, more sensitive fluorescent amyloid probes, standardization of protocols, or their mutual combination. The above mentioned procedures suggest that these methodologies could be promising ways to find a suitable biomarker, as well as a proper method for detecting, not only early stages of PD, but also indicators of disease severity and differential diagnosis.

## Abbreviations

AD	Alzheimer’s disease
α-Syn	alpha-synuclein
CSF	cerebrospinal fluid
DLB	dementia with LB
ECLIA	electro-chemiluminescence
ELISA	enzyme-linked immunosorbent assay
ENS	enteric nervous system
f-α-Syn	filamentous α-Syn
FLIM	Fluorescent Lifetime Imaging
GI	Gastrointestinal
GIT	GI tract
Hsp70	heat shock protein 70
IHC	immunohistochemical analysis
Lamp2a	lysosomal-associated membrane protein 2a
LB	Lewy bodies
LCO	luminescent conjugated oligothiophenes
L-DOPA	l-3,4-dihydroxyphenylalanine
LN	Lewy neurites
miRNAs	microRNAs
MPTP	1-methyl-4-phenyl-1,2,3,6-tetrahydropyridine
MSA	multiple system atrophy
NAC	non-amyloid-β component
o-α-Syn	oligomeric α-Syn
PMCA	cyclic amplification of misfolding proteins
p-α-Syn	phosphorylated α-Syn
PD	Parkinson’s disease
PDD	PD with dementia
PET	paraffin-embedded tissue blot
PK	Proteinase K
PLA	proximity ligation assay
PSP	progressive supranuclear palsy
PTM	posttranslational modifications
RT-QuIC	Quaking-Induced Conversion
SNpc	substantia nigra pars compacta
t-a-Syn	total α-Syn
TFEB	transcription factor EB
ThT	Thioflavin T
UTR	3′-untranslated region
